# Amphiphilic Block Copolymer Microspheres Derived from Castor Oil, Poly(ε-carpolactone), and Poly(ethylene glycol): Preparation, Characterization and Application in Naltrexone Drug Delivery

**DOI:** 10.3390/ma11101996

**Published:** 2018-10-16

**Authors:** Maria Nerantzaki, Eirini Skoufa, Kyriakos-Vasileios Adam, Stavroula Nanaki, Apostolos Avgeropoulos, Margaritis Kostoglou, Dimitrios Bikiaris

**Affiliations:** 1 Physicochemistry Laboratory of Electrolytes and Interfacial Nanosystems (PHENIX), UMR CNRS 8234, Faculty of Science and Engineering, Sorbonne University, 75252 Paris CEDEX 05, France; 2 Laboratory of Chemistry and Technology of Polymers and Dyes, Chemistry Department, Aristotle University of Thessaloniki, 54124 Thessaloniki, Greece; kva.adam@gmail.com (K.-V.A.); sgnanaki@chem.auth.gr (S.N.); 3 Laboratory of Polymeric Materials, Department of Materials Science and Engineering, University of Ioannina, Administration Building, University Campus Dourouti, 45110 Ioannina, Greece; eiriniskoufa@gmail.com (E.S.); aavger@cc.uoi.gr (A.A.); 4 Laboratory of General and Inorganic Chemical Technology, Aristotle University of Thessaloniki, 54124 Thessaloniki, Greece; kostoglu@chem.auth.gr

**Keywords:** castor oil, poly(ε-caprolactone), poly(ethylene glycol), amphiphilic block copolymers, microspheres, naltrexone, drug release

## Abstract

In the present study, the newly synthesized castor oil-derived thioether-containing ω-hydroxyacid (TEHA) block copolymers with polycaprolactone (TEHA-b-PCL), with methoxypoly(ethylene glycol) (mPEG), (TEHA-b-mPEG) and with poly(ethylene glycol) (PEG) (TEHA-b-PEG-b-TEHA), were investigated as polymeric carriers for fabrication of naltrexone (NLX)-loaded microspheres by the single emulsion solvent evaporation technique. These microspheres are appropriate for the long-term treatment of opioid/alcohol dependence. Physical properties of the obtained microspheres were characterized in terms of size, morphology, drug loading capacity, and drug release. A scanning electron microscopy study revealed that the desired NLX-loaded uniform microspheres with a mean particle size of 5–10 µm were obtained in all cases. The maximum percentage encapsulation efficiency was found to be about 25.9% for the microspheres obtained from the TEHA-b-PEG-b-TEHA copolymer. Differential scanning calorimetry and X-ray diffractometry analysis confirmed the drug entrapment within microspheres in the amorphous state. In vitro dissolution studies revealed that all NLX-loaded formulations had a similar drug release profile: An initial burst release after 24 h, followed by a sustained and slower drug release for up to 50 days. The analysis of the release kinetic data, which were fitted into the Korsmeyer–Peppas release model, indicated that diffusion is the main release mechanism of NLX from TEHA-b-PCL and TEHA-b-mPEG microspheres, while microspheres obtained from TEHA-b-PEG-b-TEHA exhibited a drug release closer to an erosion process.

## 1. Introduction

Since the pioneering studies by Bader et al. in 1984 on the use of polymeric micelles in the form of microspheres for solubilizing anti-cancer drugs, amphiphilic copolymers, which can be either block copolymers (ABCs) or graft copolymers, have been used extensively in pharmaceutical applications ranging from sustained-release technologies to gene delivery [1]. The utility of ABCs for delivery of therapeutic agents results from their unique self-assembly property in aqueous media. Such copolymers are made up of two segments of different chemical nature, both hydrophilic and hydrophobic components [2]. The hydrophobic segment forms the core of the micelle, whereas the hydrophilic segment surrounds this core as a hydrated outer shell. This core–shell structure enables polymer microspheres, such as microspheres and nanoparticles, to have potential as vehicles for drug delivery because upon micellization, the hydrophobic core regions serve as reservoirs for hydrophobic drugs, which may be loaded by chemical, physical, or electrostatic means, depending on the specific functionalities of the core-forming block and the solubilizate [3].

Naltrexone (NLX) is a potent narcotic antagonist, which is used primarily in the continued management of alcohol dependence and opioid addiction [4,5]. Despite being a relatively effective and safe treatment, the clinical management of alcohol abuse/dependence by oral NLX can be compromised due to the patient’s non-compliance with daily use of this medication. It was shown that 37% of patients discontinue daily oral NLX by 12 weeks and more than 80% discontinue use by 6 months [6]. Over the last few decades, an increasing body of research has suggested that the use of sustained release depot NLX preparations can overcome this issue [7]. Presently, there are two major sustained-release technologies for NLX release: Injectable intramuscular suspensions (Naltrel^®^, Vivitrol^®^, and Depotrex^®^), and surgically implantable pellets (Prodetoxone^®^, Wedgewood^®^, and O’Neil^®^) [8]. However, although the injectable sustained-release formulations address issues of daily oral NLX non-compliance, they still depend on patient compliance with treatment for subsequent treatments over 4 to 6 months. Subsequently, failure to return for subsequent treatment still remains a potential problem [9]. Concerning the sustained-release NLX implants, an early phase (up to 12 months post-implant) of inflammation, foreign body reaction, and fibrosis has been reported, while high cost of the implant limits its use [10].

Due to the aforementioned limitations, considerable interest has recently been shown in the design of new oral or parenteral sustained-release formulations of NLX (micelles, microspheres, and nanogels) [7,8,11]. Among these approaches, microparticulate drug delivery based on amphiphilic block copolymer microspheres (ABCs-based microspheres), has been gaining in importance. In this context, Akala et al. prepared and studied NLX-loaded poly(lactide-co-glycolide) (PLGA) microspheres with the potential of sustained drug release from 30 to 150 days [12]. Additionally, Salehi et al. developed NLX-loaded thermoresponsive micelles based on poly(N-isopropylacrylamide)-b-poly(l-lactide) (PNIPAAm-b-PLA) with block ratios of 30/70 and 70/30, capable of 5 and 18% of NLX release respectively, after 24 h [13]. The authors also suggested that in vitro release behavior could be tuned by varying the PNIPAAm to PLA ratio, since 30/70 and 70/30 formulations maintained high drug levels (45% and 10% of the initial NLX, respectively) over a period of 35 days of dissolution. Moreover, Pagar et al. employed a single emulsion solvent evaporation technique to obtain copolymer lactide-depsipeptide poly[LA-(Glc-Leu)] microspheres. In vitro release profile was characterized by an initial burst release of 49.3% from poly[LA-(Glc-Leu)] microspheres compared to 62.5% from polylactide (PLA) microspheres, while at the end of 30 days, about 80% of NLX was released [14]. To summarize, although the development of sustained release NLX products could significantly improve clinical outcomes for persons who suffer alcohol abuse/dependence, only a few micelle-based formulations have been created but suffer from instability and show very fast drug release behavior for sustained drug delivery have been proposed so far.

Consequently, despite the fact that ABCs-based microspheres seem to be a promising approach for the development of formulations with sustained NLX release ability, they need to be further stabilized. Microspheres made from conjugates of methoxypoly(ethylene glycol) (mPEG), poly(ethylene glycol) (PEG) and aliphatic polyesters such as polycaprolactone (PCL) are of particular interest because they can be useful in various controlled drug delivery applications, especially those involving relatively hydrophobic drugs [15]. We have previously reported some data on stable polymeric microspheres formed by amphiphilic triblock copolymers of PEG and poly(propylene succinate) (PPSu) with different hydrophobic/hydrophilic ratios [16]. Such microspheres can be useful in various controlled drug delivery applications, especially those involving poorly soluble drugs, including Tibolone, while they can exhibit different sizes, drug loading capacities, and drug release characteristics by varying the hydrophilic/hydrophobic ratio.

However, there might be several ways to still further enhance the drug delivery potential of ABCs-based microspheres. We have recently described the use of the castor-oil derivative TEHA, for the development of novel amphiphilic AB type (diblock) and ABA type (triblock) copolymers [17]. Such di/tri-block copolymers can be efficiently synthesized via the combination of a UV light-initiated thiol-ene “click” reaction and the following copolymerization of TEHA with ε-caprolactone (ε-CL), mPEG, or PEG. We also demonstrated that regardless of their block composition, the biorelavant yet biocompatible TEHA-based amphiphilic block copolymers are biocompatible, and therefore, they are very promising candidates for ABCs-based microparticulate delivery.

Thus, the aim of this study was to explore the potential of castor-oil derived ABCs, to be used for the development of sustained-release drug delivery systems for NLX. For this purpose, microparticles containing NLX, were prepared using TEHA-based ABCs with different hydrophobic/hydrophilic compositions: TEHA-b-PCL, TEHA-b-mPEG, and TEHA-b-PEG-b-TEHA with an oil-in-water (O/W) emulsion/solvent evaporation technique. Afterwards, all the prepared formulations were subjected to physicochemical and pharmacokinetical studies to calculate several important properties such as molecular structure, crystalline structure, particle size and morphology, encapsulation efficiency, loading capacity, and drug release behavior.

## 2. Materials and Methods

### 2.1. Materials

Naltrexone drug, undecylenic acid (10-undecenoic acid) and mPEG 2000 were purchased from Fluka (Darmstand, Germany). ε-CL (98%), 2-mercaptoethanol (99%), 2,2-dimethoxy-2-phenylacetophenone (DMPA) (99%), tin(II) 2-ethylhexanoate (Sn(Oct)_2_) (95%), PEG 1500, Poly(vinyl alcohol) (PVA), sodium chloride (NaCl), and disodium hydrogen phosphate (Na_2_HPO_4_) were supplied from Sigma-Aldrich Chemical Co. (Steinheim, Germany). Potassium dihydrogen phosphate (KH_2_PO_4_) and dichloromethane (DCM, CH_2_Cl_2_ ≥ 99.8% pure) was obtained from Chem-Lab and Merck Analytics (Kenilworth, NJ, USA), respectively. The stabilizer Tween 20 (Polysorbate 20) sorbitan monolaurate, were kindly supplied by Pharmathen S.A. (Athens, Greece). All the other reagents were of analytical grade and were used as received without further purification.

### 2.2. Synthesis of TEHA, TEHA-b-PCL, TEHA-b-mPEG, and TEHA-b-PEG-b-TEHA

**Scheme 1:**
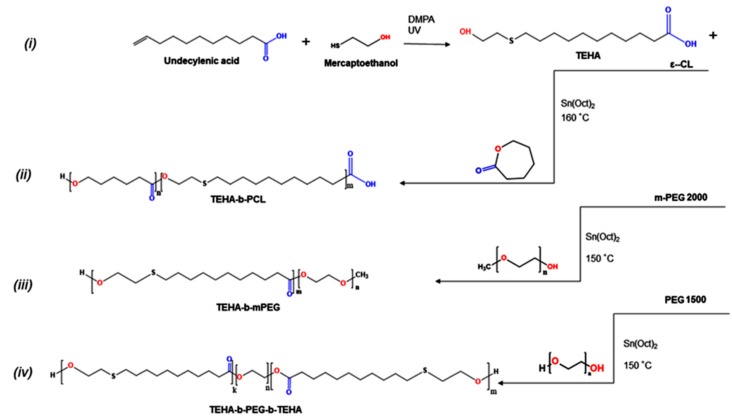
(**i**) UV-irradiated synthesis of TEHA, (**ii**) synthesis of diblock copolymer TEHA-b-PCL via ROP of ε-CL, (**iii**) synthesis of diblock copolymer TEHA-b-mPEG, and (**iv**) of triblock copolymer TEHA-b-PEG-b-TEHA using a two-step melt polycondensation method.

TEHA was freshly prepared just prior to polymerization following a previously described UV light-initiated thiol-ene “click” reaction [18]. Briefly, a solution of undecylenic acid (15 g, 81 mmol) and 2-mercaptoethanol (6.36 g, 81 mmol) in CH_2_Cl_2_ was irradiated under UV light (λ = 365 nm) in the presence of DMPA (2% TEHA/initiator molar ratio) as a photoinitiator (Scheme 1i). A TEHA-b-PCL diblock copolymer was synthesized via ring-opening polymerization (ROP) of ε-CL using TEHA as a micro-initiator and Sn(Oct)_2_, as a catalyst (initiator/catalyst molar ratio 1:1), using a synthetic strategy that has been discussed in detail elsewhere [19]. In short, TEHA and ε-CL (in molar ratio 1:5) were mixed in a 100 mL round bottom flask connected to a vacuum. The mixture was degassed with a vacuum pump (0.28 mbar) and kept at 150 °C for 1 h, under nitrogen flow and constant stirring (350 rpm). The polymerization proceeded at 160 °C for 6 h under increased mechanical stirring (720 rpm) and a high vacuum (10^−3^–10^−6^ Torr). When the polymerization was completed, the reaction product (TEHA-b-PCL) was cooled at an ambient temperature, and it was easily removed from the flask (Scheme 1ii). As previously reported, TEHA-b-mPEG diblock and TEHA-b-PEG-b-TEHA triblock copolymers were prepared through the two-stage melt polycondensation method [19]. Initially, TEHA and mPEG (molar ratio 1:0.04) or TEHA and PEG (molar ratio 1:0.05) were added to a nitrogen-purged round-bottom flask. Afterwards, the Sn(Oct)_2_-catalyzed (1% TEHA/catalyst molar ratio) esterification reaction took place under N_2_ atmosphere and constant stirring at 150 °C for 1 h. Finally, high vacuum (10^−3^–10^−6^ Torr) was applied gradually and the polycondensation was carried out for 6 h under vigorous mechanical stirring (720 rpm) (Scheme 1iii,iv).

### 2.3. Preparation of TEHA-b-PCL, TEHA-b-mPEG, and TEHA-b-PEG-b-TEHA Microspheres

An oil-in-water (O/W) emulsification and solvent evaporation technique was used for NLX microencapsulation in polyester matrices. In brief, 100 mg of polymer and 10 mg of NLX were dissolved in 5 mL of dichloromethane (DCM) and sonicated for 20 min. Afterwards, the polymer–drug solution was added in 20 mL of 0.5 wt% PVA aqueous solution and the O/W mixture was homogenized using a rotor stator stirrer (Ultra Turax IKA T18 basic, Staufen, Germany). The micronization lasted for 1 min. Afterwards, the resulting emulsion formed was gently stirred until the evaporation of the organic solvent was completed. The microspheres were collected via centrifugation (3200 rpm for 15 min), washed twice with de-ionised water, re-suspended into 5 mL of deionized water, frozen at −4 °C, and lyophilized at −104 °C. Drug free microspheres were prepared similarly, without adding NLX.

### 2.4. Characterization of Materials

#### 2.4.1. Proton Nuclear Magnetic Resonance (^1^H-NMR) Spectroscopy

^1^H-NMR spectra of copolymers were obtained using a Bruker spectrometer (Billerica, MA, USA) operating at a frequency of 400 MHz for protons. Deuterated chloroform (CDCl_3_) was used as a solvent and spectra were recorded at room temperature with tetramethylsilane (TMS) as an internal standard. The number of scans was 10 and the sweep width was 6 kHz.

#### 2.4.2. Size Exclusion Chromatography (SEC)

A size exclusion chromatograph (SEC) (Polymer Laboratories, Shropshire, UK), equipped with an isocratic pump (SpectraSystem P1000, Thermo Fisher Scientific, San Jose, CA, USA), column oven (LabAlliance, Syracuse, NY, USA) heated to 35 °C with three columns in series (PLgel 5 mm Mixed-C, 300u7.5 mm), refractive index (RI, Shodex RI-101, JM Science Inc., NY, USA), and ultraviolet absorbance (UV, SpectraSystem UV1000, Thermo Fisher Scientific, San Jose, CA, USA) detectors, and tetrahydrofuran (THF) as the eluent, was calibrated with eight polystyrene (PS) standards (Mp: 4.300 to 3.000.000 g/mol). In every case, prior to calculating the polydispersity indices (PDI) of the unknown materials as well as prior to making an estimation on the average molecular weights (Mn and Mw), a series of standard PS solutions were always tested in order to examine the accuracy of the measurements.

#### 2.4.3. X-ray Diffractometry (XRD)

XRD analysis was performed on the microsphere formulations by scanning over the 5–30° 2θ range, using a MiniFlex II diffractometer (Rigaku, model MiniFlex 600, Chalgrove, Oxford, UK) with Bragg–Brentano geometry (θ, 2θ) and a Ni-filtered Cu Kα radiation (λ = 0.154 nm).

#### 2.4.4. Fourier Transform-Infrared Spectroscopy (FTIR)

FTIR spectra were obtained using a PerkinElmer FTIR spectrometer (Dresden, Germany), model Spectrum 1000. In order to collect the spectra, a small amount of freeze-dried microparticles was mixed with KBr (1 wt% microparticles) and compressed to form tablets. The IR spectra of these tablets, in absorbance mode, were obtained in the spectral region of 450–4000 cm^−1^ using a resolution of 4 cm^−1^ and 64 co-added scans.

#### 2.4.5. Differential Scanning Calorimetry (DSC)

A Perkin–Elmer, Pyris Diamond Differential Scanning Calorimeter (Dresden, Germany), calibrated with indium and zinc standards, was employed. A sample of about 10 mg was placed in a scaled aluminum pan and heated from 30 °C to 200 °C at a heating rate of 10 °C/min. The sample was held at that temperature for 5 min in order to erase any thermal history. The samples were cooled from the melt down to 30 °C at a cooling rate of 10 °C/min and the subsequent heating scans from 30 °C to 200 °C at a heating rate of 5 °C/min were recorded.

#### 2.4.6. Scanning Electron Microscopy (SEM)

The morphology of the prepared microspheres was studied using scanning electron microscopy (SEM) (JEOL JMS-840 apparatus, Tokyo, Japan). The samples were covered with a carbon coating in order to provide good conductivity of the electron beam. Operating conditions were: Accelerating voltage 20 kV, probe current 45 nA, and counting time 60 s.

#### 2.4.7. Evaluation of Drug Encapsulation

Quantitative analysis was performed using a Shimadzu high pressure liquid chromatography (HPLC, Shimadzu Scientific Instruments, Tokyo, Japan) prominence system consisting of a degasser (DGU-20A5, Tokyo, Japan), a liquid chromatograph (LC-20 AD, Shimadzu Scientific Instruments, Tokyo, Japan), an auto sampler (SIL-20AC, Shimadzu Scientific Instruments, Tokyo, Japan), a UV/Vis detector (SPD-20A, Shimadzu Scientific Instruments, Tokyo, Japan) and a column oven (Athena C18, Shimadzu Scientific Instruments, Tokyo, Japan). A modified previously validated method was used for the analysis [13]. In detail, a CNW Technology Athena C18, (250 mm × 4.6 mm and 5 µm internal diameter) at column temperature of 40 °C chromatographic work station (CSW)was used for regression analysis and data acquisition. Concentration determination was performed using an HPLC-UV apparatus at 280 nm and was based on a previously created calibration curve. Flow rate was 1 mL/min and the injection volume was 20 μL/min. The calibration curve was created by diluting a stock aqueous solution of 400 ppm naltrexone to concentrations 0.5, 1, 2.5, 5, 10, 20, 30 and 50 ppm using the mobile phase: A 10 mM phosphate buffer (pH was adjusted to 3.5 using H_3_PO_4_) with acetonitrile in a ratio of 80:20. Each sample was measured in triplicate. The percentage yield, drug loading, and drug entrapment efficiency of microspheres were calculated from the following equations, respectively:

(1) $$\mathrm{Yield}\;\mathrm{of}\;\mathrm{microspheres}\;\left(\%\right)=\frac{\left(\mathrm{weight}\;\mathrm{of}\;\mathrm{microspheres}\right)}{\left(\mathrm{weight}\;\mathrm{of}\;\mathrm{polymer}\;\mathrm{and}\;\mathrm{drug}\;\mathrm{fed}\;\mathrm{initially}\right)}\times 100$$

(2) $$\mathrm{Drug}\;\mathrm{loading}\;\left(\%\right)=\frac{\left(\mathrm{weight}\;\mathrm{of}\;\mathrm{drug}\;\mathrm{in}\;\mathrm{microspheres}\right)}{\left(\mathrm{weight}\;\mathrm{of}\;\mathrm{microparticles}\right)}\times 100$$

(3) $$\mathrm{Entrapment}\;\mathrm{efficiency}\;\left(\%\right)=\frac{\left(\mathrm{weight}\;\mathrm{of}\;\mathrm{drug}\;\mathrm{in}\;\mathrm{microspheres}\right)}{\left(\mathrm{weight}\;\mathrm{of}\;\mathrm{initially}\;\mathrm{used}\;\mathrm{drug}\right)}\times 100$$

#### 2.4.8. In Vitro Release Profile

NLX-loaded microspheres were sealed with cellulose dialysis membranes and were put in the sealed glass vials which were filled with 20 mL of 0.01 mol/L phosphate buffer solution (pH 7.4) containing 0.02% NaN_3_ and 0.04 polysorbate 20 (Tween 20). Three vials were set for each type of naltrexone microspheres. All the vials were incubated with horizontal oscillation (80 cycles/min) at 37 °C. Solutions were substituted completely with fresh ones at predetermined intervals. Substituted solutions at each time point were filtered through 0.45μm filter and sampled on the HPLC column.

## 3. Results and Discussion

### 3.1. Synthesis and Characterization of Amphiphilic TEHA-Based Block Copolymers

**Figure 1:**
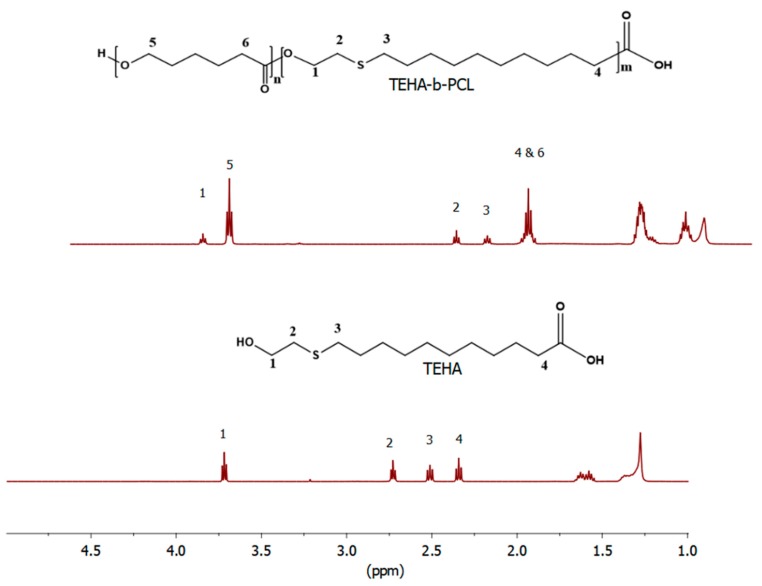
^1^H-NMR spectra of TEHA and TEHA-b-PCL diblock copolymer.

In this study, TEHA was synthesized following a previously described UV-induced thiol-Michael addition reaction, between 2-mercaptoethanol and 10-undecylenic acid (Scheme 1i). The completion of the thiol–ene reaction was confirmed by the absence of the vinylic and allylic hydrogen signals [18,20] (around 5.00 and 2.00 ppm, respectively) in the ^1^H-NMR spectrum of TEHA (Figure 1). In addition to the total conversion of the double bonds, new ^1^H chemical shifts of protons attached to the carboxylic and hydroxyl groups were detected in the spectrum of TEHA at 2.34 ppm and 3.72 ppm, respectively (Figure 1, H_1_, H_4_), while the methylene protons linked to sulfur appeared as two triple peaks at 2.73 ppm and 2.51 ppm (Figure 1, H_2_, H_3_).

The ^1^H-NMR spectrum of the (AB type) diblock copolymer TEHA-b-PCL, which was synthesized via ROP of ε-CL using TEHA as a micro-initiator and Sn(Oct)_2_ as a catalyst, is displayed together with the spectrum of TEHA in Figure 1. As shown, TEHA-b-PCL has one multiple peak at 2.30 ppm which corresponds to the –CH_2_CO– protons in PCL and TEHA units (H_4_,_6_), and two triple peaks at 2.55 and 2.75 ppm, which can be assigned to the methylene protons linked to the sulfur atom (–CH_2_–S–) of TEHA block (H_2_, H_3_) (Scheme 1ii). More importantly, in the spectrum of TEHA-b-PCL, the total conversion of the thioether functional groups was confirmed by the existence of shifts typical of –CH_2_–OH and CH_2_–O– proton signals at 4.20 and 4.05 ppm, respectively [17].

**Figure 2:**
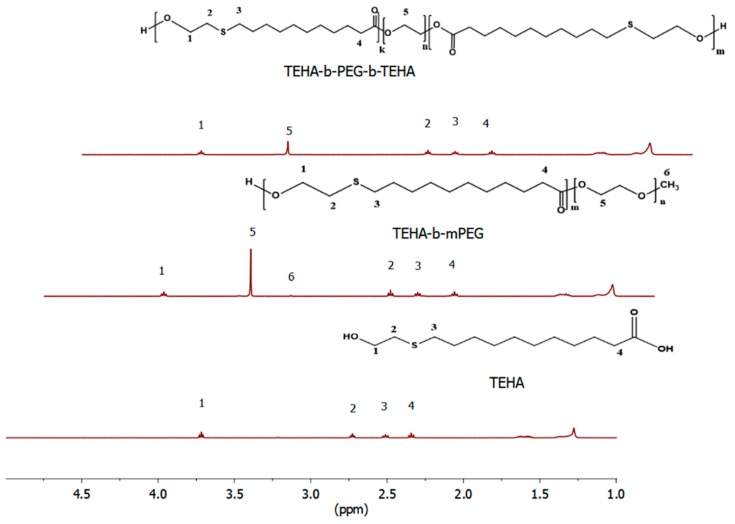
^1^H-NMR spectra of TEHA, TEHA-b-mPEG diblock, and TEHA-b-PEG-b-TEHA triblock copolymers.

To obtain the (AB type) di-block and (ABA type) tri-block copolymers TEHA-b-mPEG and TEHA-b-PEG-b-TEHA, a simple ester bond formation strategy between the carboxyl groups of TEHA and the hydroxyl groups of mPEG 2000 and PEG 1500 was used, as given in Scheme 1iii,iv. The structure and composition of the synthesized block copolymers was determined by ^1^H-NMR and the resulting spectra are presented in Figure 2, together with the spectrum of TEHA. For the diblock copolymer TEHA-b-mPEG, the methylene protons linked to sulfur (–CH_2_-S–) gave two triple peaks, which were observed at 2.55 and 2.75 ppm and those linked to carbonyl group (–CH_2_CO–) and one triple peak at 2.20 ppm (Figure 2, H_2_, H_3_, H_4_). Moreover, the signal of the protons linked to the hydroxyl group (–CH_2_–OH) was recorded at a higher chemical shift than for TEHA and appeared as a triple peak at 4.23 ppm (Figure 2, H_1_). In addition to the higher chemical shift, the appearance of a new peak corresponding the methylene protons of methoxy group (–CH_2_OCH_3_) at 3.65 ppm, further confirmed the successful synthesis of the TEHA-b-mPEG diblock copolymer (Figure 2, H_5_) [20].

Correspondingly, the ^1^H-NMR spectrum of TEHA-b-PEG-b-TEHA copolymer exhibited peak characteristics of both PEG and TEHA units. The signals assigned to the methylenes of (–CH_2_–S–) and (–CH_2_CO–) groups were recorded at 2.55 ppm, 2.75 ppm (Figure 2, H_2_, H_3_), and 2.30 ppm (Figure 2, H_4_), respectively. In Figure 2, the signals corresponding to the (–CH_2_–OH) group come at a noticeably higher chemical shift than in TEHA and are found at 4.23 ppm, while those assigned to the methylene protons in methoxy group of PEG (–CH_2_OCH_3_) appear as a new peak at 3.65 ppm, further supporting the fact that the TEHA-b-PEG-b-TEHA tri-block copolymer was successfully synthesized (Figure 2, H_1_, H_5_).

**Figure 3:**
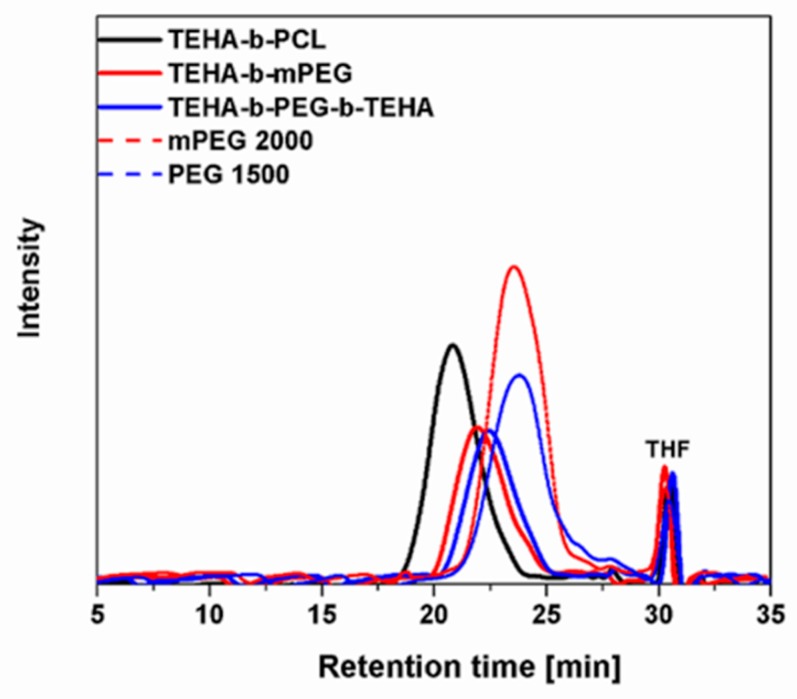
SEC traces of TEHA-b-PCL, TEHA-b-mPEG, and TEHA-b-PEG-b-TEHA block copolymers, compared to the peaks obtained for mPEG 2000 and PEG 1500.

SEC chromatography further confirmed the successful synthesis of TEHA-b-PCL, TEHA-b-mPEG, and TEHA-b-PEG-b-TEHA block copolymers since the corresponding SEC-traces, which are presented together with the traces of mPEG 2000 and PEG 1500 in Figure 3, showed a monomodal distribution [21]. More specifically, all the SEC traces of the block polymers were shifted to higher elution times, in comparison with the traces recorded for the initial mPEG 2000 and PEG 1500 monomers.

Table 1 shows the molecular weights and polydispersity indices (PDI) of the TEHA-based block copolymers. The calculated number-based average molecular weights (Mn) of TEHA-b-PCL, TEHA-b-mPEG, and TEHA-b-PEG-b-TEHA were in good accordance with the initial feed ratios and were found to be 9800 g/mol, 4800 g/mol, and 2.500 g/mol respectively. In addition, small polydispersity index (PDI) values close to 1 were detected. These results indicated that no transesterification and/or backbiting reactions occurred during the copolymerization [22].

### 3.2. Evaluation of Drug-Loaded Microspheres Prepared from Amphiphilic TEHA-Based Block Polymers

#### 3.2.1. Drug Loading and Morphology of Prepared Microspheres

In the present study, NLX-loaded microspheres were prepared by using the O/W solvent evaporation method. This method was selected because the drug and the polymers were soluble in DCM. The percentage yields, drug loading capacity (DL), and entrapment efficiencies (EE) for NLX-loaded microspheres prepared from the amphiphilic copolymers TEHA-b-PCL, TEHA-b-mPEG, and TEHA-b-PEG-b-TEHA are shown in Table 2. The maximum percentage EE was found to be 25.9% for the microspheres obtained from copolymer TEHA-b-PEG-b-TEHA, which also had the highest yield and drug loading compared to the other copolymers. TEHA-b-PCL also showed a high DL percentage and yield of 1.19% ± 0.6% and 41.5% ± 2%, respectively. This low drug loading was expected due to the high hydrophobicity of the NLX drug [15,22].

In general, several parameters, such as nature of the hydrophilic (corona) and hydrophobic (core) forming block, the length of the core, and total copolymer molecular weight are some parameters affecting the DL, as well as the nature and length of the corona forming block [23,24]. For a block copolymer drug delivery vehicle system with a constant length of the hydrophilic segment, it was expected that an increase in the length of core forming block raised the partition coefficient for the solubilizate [25]. Therefore, it is suggested here that the copolymers with longer hydrophobic chains self-assembled into micelles in the form of microspheres with a more compact inner core, reducing the aggregation possibility of drug-loaded microspheres and improving the DL through increasing the hydrophobic interaction between the drug and the copolymer [26].

**Figure 4:**
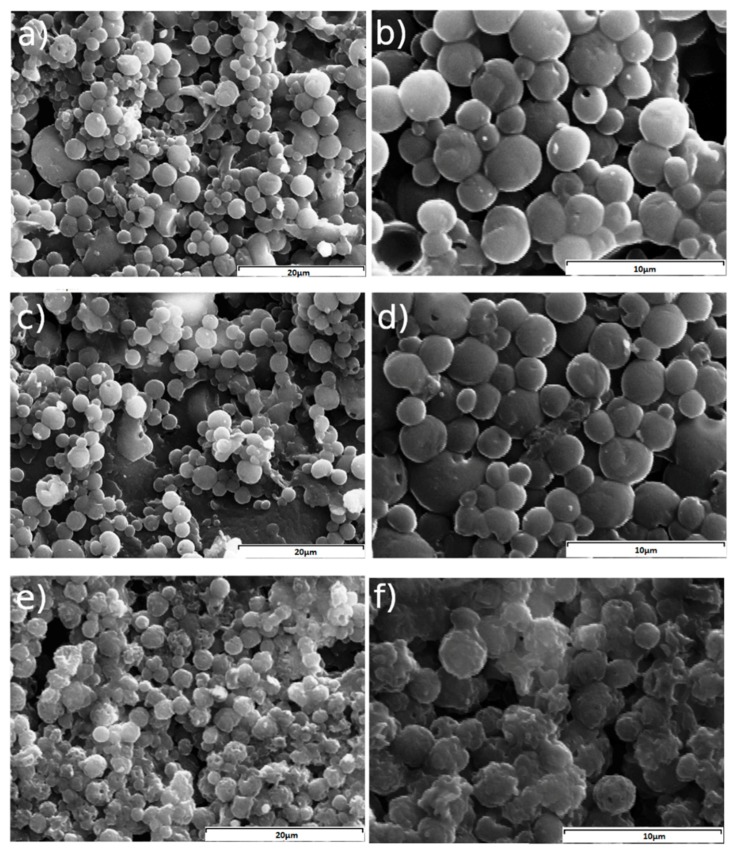
SEM images of (**a**,**b**) NLX-loaded/TEHA-b-PCL, (**c**,**d**) NLX-loaded TEHA-b-mPEG, and (**e**,**f**) NLX-loaded/TEHA-b-PEG-b-TEHA microspheres at low (left) and high (right) magnification.

The morphology of the prepared microspheres was another determining factor of drug vehicle efficacy and thereby parameters affecting the micelle size are required to be revealed in order to prepare long circulating microparticulate drug carriers [27]. In this study, SEM was used to determine the size, shape, and surface morphology of the NLX-loaded microspheres. As shown in Figure 4, well-defined NLX-loaded microspheres with a uniform spherical shape were prepared by using a simple O/W emulsion technique. More particularly, in Figure 4a–d it can be seen that NLX-loaded microspheres prepared using TEHA-b-PCL and TEHA-b-mPEG were spherical in form, mostly having smooth surfaces, while NLX-loaded microspheres prepared uisng TEHA-b-PEG-b-TEHA had a spherical shape and comparatively rough surfaces (Figure 4e,f).

Another crucial property that should be considered while preparing drug delivery vehicles is the size of microspheres. Those microspheres that are smaller than 200 nm in size are less susceptible to rapid removal from the circulatory blood stream by the reticuloendothelial system and those smaller than 5 µm are able to enter small capillaries [28]. SEM images in Figure 4 indicate that desired NLX-loaded spherical microspheres with a mean particle size of 5–10 µm were obtained at a stirring speed of 3200 rpm; no clear differences were seen between the different used copolymers. Generally, the smaller the mean particle size, the shorter the diffusion path for drug release, and thus, the faster the release rate and vice versa. Consequently, controlling the particle size provides a means of tuning drug release profiles [29].

#### 3.2.2. FTIR, XRD and DSC Analysis of Microparticles

**Figure 5:**
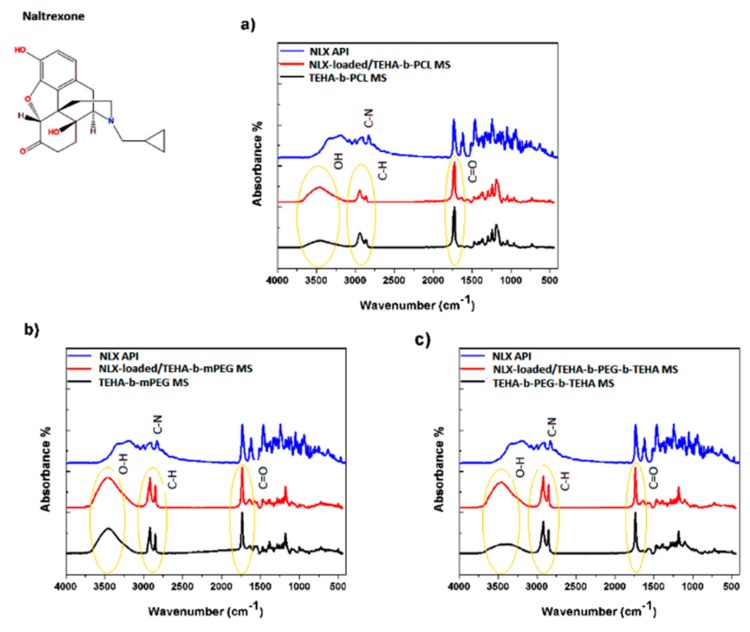
FTIR spectra of NLX in comparison with drug-free and drug loaded microspheres prepared from amphiphilic TEHA-based polymers: (**a**) TEHA-b-PCL; (**b**) TEHA-b-mPEG and (**c**) TEHA-b-PEG-b-TEHA.

Figure 5 depicts the FTIR spectra of NLX in comparison with drug-free and drug-loaded microspheres prepared from the amphiphilic TEHA-based polymers TEHA-b-PCL, TEHA-b-mPEG, and TEHA-b-PEG-b-TEHA. The FTIR spectra of NLX presents absorption bands at 3000–3100, 1655, 1558, and 1457 cm^−1^ assigned to the benzene rings, at 1456 cm^−1^ and 1100 cm^−1^ due to C–C stretching bands and C-O respectively, a strong peak at 1765 cm^−1^ due to its >C=O group, and a broad peak at 3450 cm^−1^ due to the existence of –OH groups [30]. The FTIR spectra of all NLX-free microspheres showed the characteristic peaks commonly found in the polymer chain backbone: A broad band at 3600–3300 cm^−1^, which was assigned to O–H stretching, the stretching vibration band of C=O at 1700–1680 cm^−1^, and the C–H stretching vibration peak at 3000–2900 cm^−1^ [31]. Moreover, the ester vibrations showed characteristic peaks at 1268–1020 cm^−1^. The FTIR spectra for NLX-loaded microspheres were almost identical to the spectra obtained for NLX-free microspheres, as shown in Figure 5, suggesting the absence of a chemical interaction between drug and copolymers.

**Figure 6:**
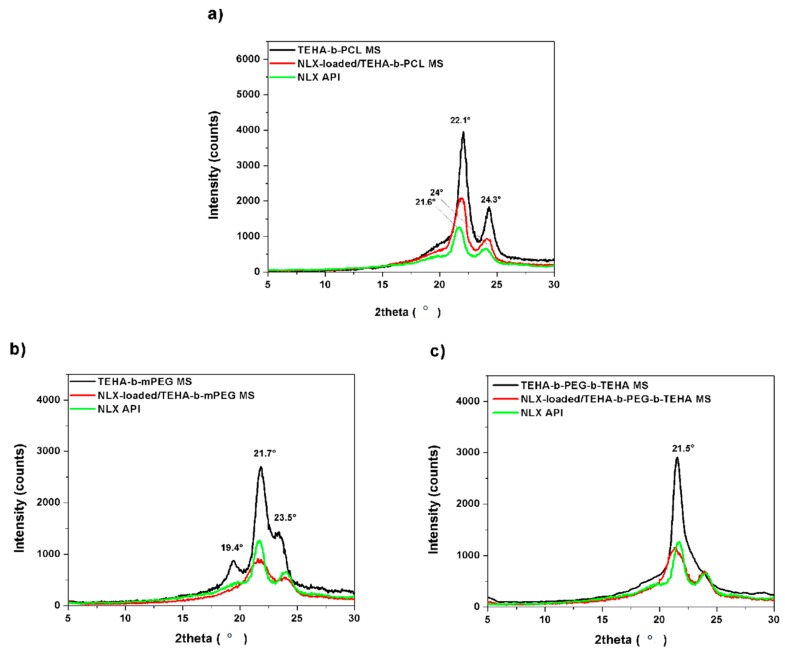
XRD patterns of NLX API in comparison with NLX-free and NLX-loaded microspheres prepared from amphiphilic TEHA-based copolymers: (**a**) TEHA-b-PCL, (**b**) TEHA-b-mPEG, and (**c**) TEHA-b-PEG-b-TEHA.

XRD analysis was used to study the crystal structure of the drug in the formed microspheres. NLX showed the characteristic intense peaks at 2θ 21.6° and 24° due to its crystalline nature [32]. However, in XRD curves for NLX-loaded/TEHA-b-PCL MS and NLX-loaded/TEHA-b-mPEG MS, presented in Figure 6a,b, could not be observed because they overlapped with the peaks assigned to the polymer chains of TEHA-b-PCL (2θ 22.1° and 24.3°) and TEHA-b-mPEG (2θ 21.7° and 23.5°). In the case of NLX-loaded/TEHA-b-PEG-b-TEHA MS (Figure 6c), the sharp peak of TEHA-b-PEG-b-TEHA was recorded at 2θ 21.5°, while a weak peak corresponding to NLX appeared at 2θ 21.5°, suggesting that the crystalline state of NLX was reduced or NTX was in its amorphous state. This decrease in crystallinity was probably due to the inhibition effect of the copolymer on the drug crystallization procedure [33]. It is worth mentioning that for all NLX-loaded MS, the signals assigned to TEHA-b-PCL, TEHA-b-mPEG, and TEHA-b-PEG-b-TEHA copolymer chains were weak compared with the blank microsphere. This result indicates that the crystalline state of the copolymers was also reduced during the drug encapsulation process [34].

**Figure 7:**
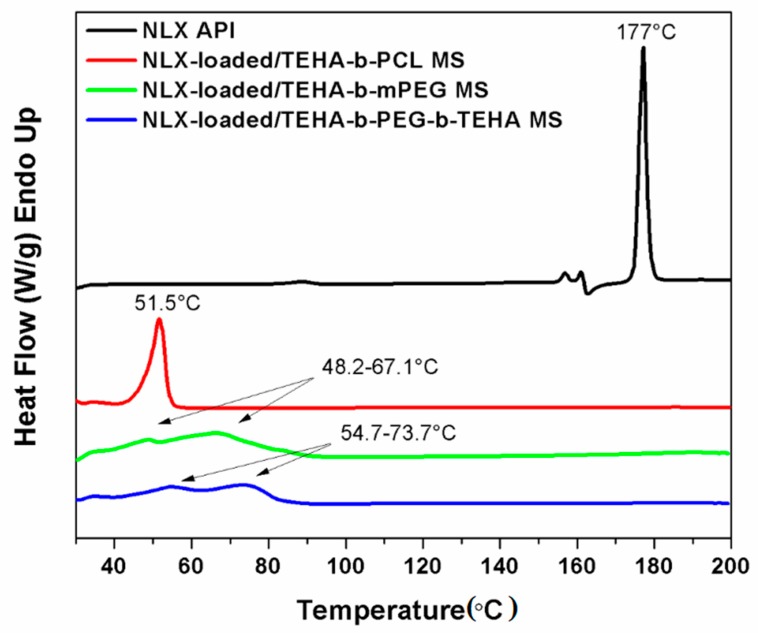
DSC thermograms of NLX API, NLX-loaded TEHA-b-PCL, NLX-loaded/TEHA-b-mPEG, and NLX-loaded TEHA-b-PEG-b-TEHA microspheres.

DSC curves of NLX and NLX-loaded TEHA-b-PCL, TEHA-b-mPEG, and TEHA-b-PEG-b-microspheres are shown in Figure 7. NLX showed an endothermic peak near 177 °C due to its melting point [32], while for the NLX-loaded microspheres, endothermic peaks were not recorded, suggesting that the drug was encapsulated in the amorphous state and that it was distributed in the polymer matrix. In agreement with results of previous DSC studies [17], the endothermic peaks of TEHA-b-PCL, TEHA-b-mPEG and TEHA-b-PEG-b-TEHA were recorded at 51.5, 48.2–67.1 (double peak) and 54.7–73.7 °C (double peak), respectively.

#### 3.2.3. In Vitro Release Properties

Release of drug molecules from the micellar vehicle is as important as loading of the microspheres. The release rate can be considered as diffusion and affected from the micelle stability and biodegradability of the copolymer micelle [35]. If the micelle is stable enough and biodegradability rate is low, the release rate of drug molecules from the micelle core is influenced by many factors such as the strength of the interaction between the core forming block and drug molecules, the physical state of the micelle core, the amount of drug molecules loaded, the molecular volume of drug molecules, and the length of the core forming block and the localization of drug molecules within the micelle [36]. Most of these factors also affect the loading capacity of the micelle and could have a reverse effect on it. For this reason, the optimum value for these factors should be figured out while designing a new drug delivery vehicle [37].

**Figure 8:**
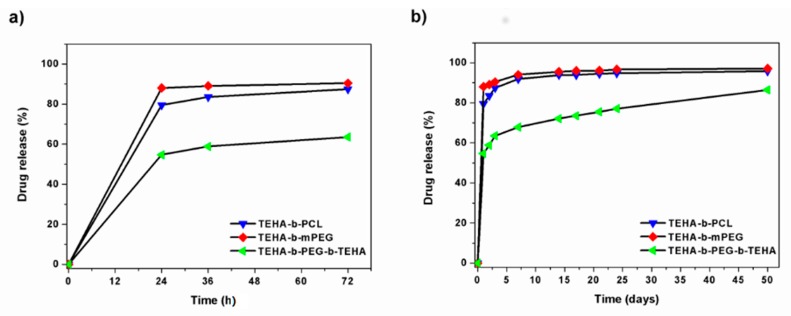
Percentage of release of NLX from microspheres prepared from amphiphilic TEHA-based copolymers at different time intervals: (**a**) 24, 48 and 72 h, and (**b**) 1, 2, 3, 7, 14, 17, 21, 24 and 50 days.

In this study, the in vitro release behavior of micelle-encapsulated NLX in phosphate buffered saline (PBS) (pH 7.4) at 37 °C was studied using a dialysis method, and the results are shown in Figure 8. The in vitro drug release study indicated that the molecular characteristics of di/triblock copolymers used in this study, affected the release rate of drug from microspheres. More specifically, TEHA-b-PCL and TEHA-b-mPEG micelle-encapsulated NLX was observed to be rapidly released and reached its peak of 80% and 89% of the total in the first 24 h. In comparison, for TEHA-b-PEG-b-TEHA micelle-encapsulated NLX, the burst effect was less and about 55% of NLX was released in 72 h. A three-step drug release profile is shown in Figure 8b. A large initial burst release occurred within the first 5 days, followed by a long period of matrix-diffusion kinetics, and then a very slow release rate. NLX loaded in TEHA-b-PEG-b-TEHA copolymer showed a lower burst release compared with the other two copolymers, and there was a sustained release up until 50 days. The higher release rate of NLX from TEHA-b-PCL microspheres should be attributed to the lower melting point that this copolymer has. This was proved in our previous studies in aliphatic polyesters and copolymers [38,39]. A melting point of an aliphatic polyester close to body temperatures gives the highest rates. This also explains the lowest release rate of NLX from the TEHA-b-PEG-b-TEHA copolymer, which according to recorded DSC thermographs, has the highest melting point from all copolymers.

**Figure 9:**
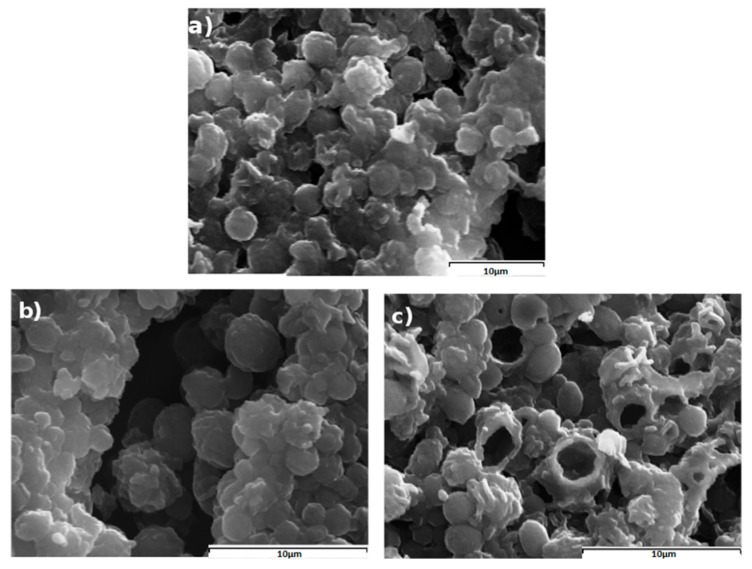
SEM images of (**a**) NLX-loaded/TEHA-b-PCL; (**b**) NLX-loaded TEHA-b-mPEG; (**c**) NLX-loaded/TEHA-b-PEG-b-TEHA microspheres after 50 days of dissolution.

The in vitro release results indicated that NLX could be slowly released from microspheres in a sustained manner in vitro. The sustained release of NLX from the microspheres might be due to two reasons: (1) The diffusion of NLX from the microspheres to the medium, and (2) Degradation and hydrolysis of microspheres induced release of NLX to the medium. Figure 9 presents the morphology of the microspheres after 50 days of dissolution. It is clear there the surface of all microspheres have been changed after drug dissolution, and in all cases, the surface became rough and the regular spherical shape that was observed in initial microspheres has been lost. Furthermore, the internal morphology in TEHA-b-PEG-b-TEHA confirms the presence of cavities, which shows that the drug passed from the polymer matrix into the external environment and some erosion also took place. Therefore, it seems that diffusion was the main mechanism for TEHA-b-PCL and TEHA-b-mPEG copolymers while diffusion and erosion could be the release mechanism for NLX from TEHA-b-PEG-b-TEHA copolymers [40].

#### 3.2.4. Drug Release Kinetics

**Figure 10:**
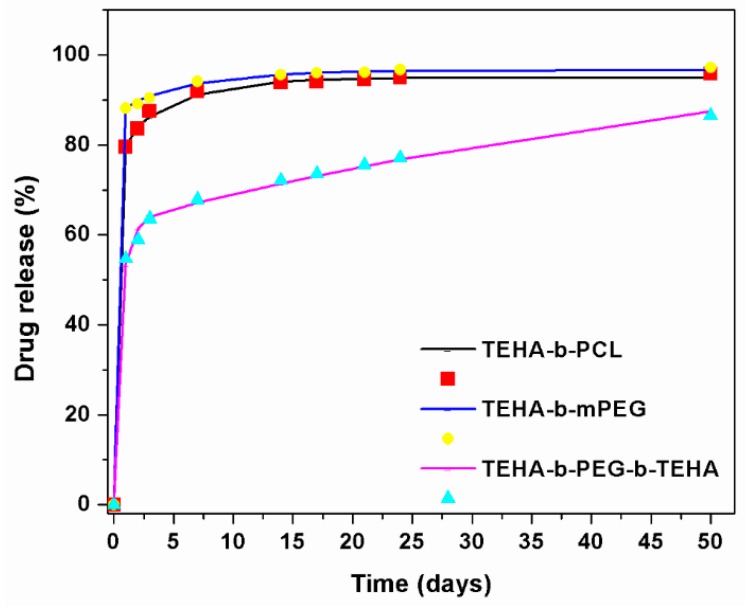
Comparison between model and experimental data of NLX release.

In the present section the drug release kinetics data will be analyzed based on the corresponding theories. The scope of such an analysis is two-fold: At first to create a mathematical model for the process and second to extract information on the structure of the drug-particle system. In general, the drug release from polymeric matrices can be due to two different physical processes: (i) Diffusion of the drug in the polymer matrix and (ii) Polymer erosion leading to the release of the drug located at the eroded polymer portion. The intense mixing in the present experiments ensured that the resistance to mass transfer of drugs from the polymer surface to the bulk liquid could be neglected. The experimental kinetics data are restricted to the region of completion (high total release) so the Peppas–Korsmayer generalized diffusion model, which can be applied for release up to 60%, cannot be used here [41]. As it has been already discussed in a previous section, the dominant release mechanism for copolymers TEHA-b-PCL and TEHA-b-mPEG appears to be diffusion. Let us first consider the diblock copolymer TEHA-b-PCL. The first order kinetics model, which is an approximation of the linear (Fickian) diffusion mechanism, was tested as a starting point. The fitting process led to failure since the matching to the first data point corresponded to a release process ending at 3 days. The next step was to consider the exact solution for Fickian diffusion in spherical coordinates (since the polymer particles had an approximately spherical shape). This solution has the form [42]:

(4) $$M=M_{o}(1-\frac{6}{\pi^{2}}\sum_{i=1}^{\infty }\frac{1}{i^{2}}\exp(-Di^{2}\pi^{2}t/R^{2}))$$

where M is the released fraction, M_o_ is the final released fraction, t is time (in days), R is the spherical particle radius, and D is the diffusion coefficient of the drug in the polymer matrix. The fitting procedure did not improve the situation met by the first order kinetic model. Therefore, a simple diffusion process could not describe the experimental data. There are several possible explanations for this behavior. The most straightforward are the non-uniform distribution of drugs in the particles and/or the existence of several regions in the particles with different diffusion coefficients. In the absence of additional information, the second scenario is employed here since it admits much easier manipulation. By considering the deviation between the diffusion model and the data, it was found that a simple exponential may be used to fill the gap. This corresponds to a fraction of the drug that diffused with a different diffusion coefficient. The model equation in this scenario is:

(5) $$M=M_{1}(1-\frac{6}{\pi^{2}}\sum_{i=1}^{\infty }\frac{1}{i^{2}}\exp(-D_{1}i^{2}\pi^{2}t/R^{2}))+U(t-1)M_{2}(1-\exp(-A(t-1)))$$

where M_1_ and M_2_ are the fractions of total drug having diffusion coefficients D_1_ and D_2_, respectively. The fitting coefficient A is related to the diffusivity D_2_ through the so-called linear driving force formula: A = 15D_2_/R^2^ [43]. The function U is the so-called step function taking the values zero and one for negative and positive arguments, respectively. The fact that the second diffusion process started at t = 1 implies that there was a lack of this type of drug in the external regions of the particles. Assuming a (volume) average particle radius of 2 μm as resulted from the SEM pictures, and successfully fitting Equation (5) to the experimental data, leads to the following parameter values: M_1_ = 0.83, M_2_ = 0.13, D_1_ = 1.4 × 10^−17^ m^2^/s, and D_2_ = 6.17 × 10^−19^ m^2^/s. The comparison between the model and experimental data is shown in Figure 10 (M had to be multiplied by 100 to become a percentage release). The maximum deviation between the model and experiment was 1%, which is quite acceptable given the experimental uncertainty. However, the release data showed that there was a small but detectable erosion process (of the order of 1% per 30 days). This erosion process should be included in the model if even smaller deviations from the data were pursued or predictions for time larger than 50 days were required.

The diblock copolymer TEHA-b-mPEG exhibited the same exact behavior with TEHA-b-PCL. The same Equation (5) was used to fit the data with the corresponding comparison shown in Figure 10. The fractions M_1_ = 0.89 and M_2_ = 0.08 of the drug were diffused with diffusion coefficients D_1_ = 2.1 × 10^−17^ m^2^/s and D_2_ = 4.9 × 10^−19^ m^2^/s, respectively. The diffusion coefficients were comparable to those of TEHA-b-PCL. The remaining (not diffusing) 3% of the drug was released through erosion with a rate of 1% per 50 days.

The release data for the triblock copolymer TEHA-b-PEG-b-TEHA shows that there was a large contribution from erosion. The form of the release curve suggests that there was a fraction of the drug release by diffusion alone and another fraction released by erosion alone (no diffusing). The diffusion process ceased before the erosion substantially reduced the size of the particle such that the two release mechanisms could be assumed to act independently. The polymer erosion occurred with a constant rate, let us denote it k (units: length/time) [44]. For a slab geometry, this corresponds to a constant release rate (zero order kinetics). In the case of spherical geometry with a large erosion extent, the situation was somewhat different. The radius of the particle at time t will be R_1_ = R − kt which means that the fraction of non-diffused drug remaining in the particle in this time will be (R − kt)^3^/R^3^. Taking into account the above arguments, the following model equation results for this copolymer:

(6) $$M=M_{1}(1-\frac{6}{\pi^{2}}\sum_{i=1}^{\infty }\frac{1}{i^{2}}\exp(-Di^{2}\pi^{2}t/R^{2}))+(1-M_{1})(1-{\left(1-kt/R\right)}^{3})$$

where M_1_ is the fraction of the drug undergoing diffusion. The rest of the drug (fraction 1 − M_1_) is non-diffusing and it is released during particle erosion. The comparison between model and experimental data is shown in Figure 10. The fitting parameters were (diffusing drug fraction) M_1_ = 0.625, (diffusion coefficient) D = 0.63 × 10^−17^ m^2^/s, and (erosion rate) k/R = 0.006 day^−1^. One could unify the observed behavior for all three copolymers saying that there were two modes of diffusion plus erosion release mechanisms. The erosion rate was actually zero for the first two copolymers and the second mode diffusion coefficient was zero for the third copolymer.

## 4. Conclusions

In conclusion, this study confirmed that the castor oil derived copolymers: TEHA-b-PCL, TEHA-b-mPEG, and TEHA-b-PEG-b-TEHA, which could be obtained using a one-pot synthetic procedure involving TEHA, ε-CL, mPEG, and PEG, could be efficiently used for the preparation of amphiphilc block copolymer microspheres. The results of the study suggest that several factors, such as Mw and composition of the amphiphilic copolymers, significantly affect the morphological, physicochemical, and pharmaceutical characteristics of the resulting NLX-loaded microspheres, which were prepared with a desirable size of 5–10 μm and different entrapment efficiencies, which varied from 7.6 ± 0.9% to 25.9 ± 1.4%. All NLX-loaded microspheres showed an initial burst effect to provide the loading dose of the drug, followed by sustained release for 50 days, suggesting that these delivery systems can be potential carriers for long-acting controlled drug delivery. Furthermore, analysis of release kinetics data shows that the release mechanism was multiple mode Fickian diffusion for TEHA-b-mPEG and TEHA-b-PCL NLX-loaded microspheres. On the contrary, polymer erosion was comparable with the diffusion contribution for the release kinetics for microspheres prepared using the TEHA-b-PEG-b-TEHA copolymer.

## Figures and Tables

**Table 1:** Molecular characteristics of TEHA-b-PCL, TEHA-b-mPEG, and TEHA-b-PEG-b-TEHA block copolymers.

Sample	Feed Molar Ratio ^1^	Mn ^2^ (g/mol)	Mw ^2^ (g/mol)	PDI ^2^
TEHA-b-PCL	TEHA:ε-CL, 1:5	9800	14.800	1.55
THEA-b-mPEG	TEHA:mPEG, 1:0.05	4.800	6.700	1.40
TEHA-b-PEG-b-TEHA	TEHA:PEG, 1:0.04	2.500	3.675	1.47

^1^ Hydrophylic/hydrophobic block ratio. ^2^ Determined using SEC with THF as the eluent and polystyrene as standards.

**Table 2:** Percentage yield, drug loading and encapsulation efficiency of prepared microspheres.

Sample	Yield (%)	Drug Loading (%)	Encapsulation Efficiency (%)
NLX-loaded/TEHA-b-PCL	41.5 ± 2.1	1.19 ± 0.6	25.6 ± 1.5
NLX-loaded/THEA-b-mPEG	32.3 ± 3.2	0.27 ± 0.07	7.6 ± 0.9
NLX-loaded/TEHA-b-PEG-b-TEHA	89.9 ± 5.2	2.71 ± 0.5	25.9 ± 1.4
